# Evaluation of Biofilm Formation and Prevalence of Multidrug-Resistant Strains of *Staphylococcus epidermidis* Isolated from Neonates with Sepsis in Southern Poland

**DOI:** 10.3390/pathogens10070877

**Published:** 2021-07-11

**Authors:** Iwona Skiba-Kurek, Paweł Nowak, Joanna Empel, Magdalena Tomczak, Joanna Klepacka, Iwona Sowa-Sierant, Iwona Żak, Bartosz Pomierny, Elżbieta Karczewska

**Affiliations:** 1 Department of Pharmaceutical Microbiology, Faculty of Pharmacy, Jagiellonian University Medical College, Medyczna 9 Street, 30-688 Krakow, Poland; iwona.skiba@uj.edu.pl (I.S.-K.); p.nowak@uj.edu.pl (P.N.); 2 Department of Epidemiology and Clinical Microbiology, National Medicines Institute, Chełmska 30/34 Street, 00-725 Warsaw, Poland; j.empel@nil.gov.pl (J.E.); m.tomczak@nil.gov.pl (M.T.); 3 Department of Clinical Microbiology, University Children’s Hospital of Krakow, Wielicka 256 Street, 30-663 Krakow, Poland; jklepacka@usdk.pl (J.K.); isowa@usdk.pl (I.S.-S.); izak@usdk.pl (I.Ż.); 4 Department of Toxicology, Faculty of Pharmacy, Jagiellonian University Medical College, Medyczna 9 Street, 30-688 Kraków, Poland; bartosz.pomierny@uj.edu.pl

**Keywords:** *Staphylococcus epidermidis*, antibiotic resistance, MRSE, biofilm, *ica* genes, neonatal septicemia, nosocomial infections

## Abstract

*Staphylococcus epidermidis* strains play an important role in nosocomial infections, especially in the ones associated with biofilm formation on medical devices. The paper was aimed at analyzing the mechanisms of antibiotic resistance and confirming the biofilm-forming ability among *S. epidermidis* strains isolated from the blood of hospitalized newborns. Genetic analysis of resistance mechanism determinants included multiplex PCR detection of *mecA*, *ermA*, *ermB, ermC*, *msrA*, and *mef* genes. Biofilm analysis comprised phenotypic and genotypic methods including Christensen and Freeman methods and PCR detection of the *ica*ADB gene complex. Among the tested *S. epidermidis* strains, 89% of the isolates were resistant to methicillin, 67%—to erythromycin, 53%—to clindamycin, 63%—to gentamicin, and 23%—to teicoplanin, while all the strains were susceptible to vancomycin and linezolid. The *mecA* gene was detected in 89% of the isolates, the *ermC* gene was the most common and present among 56% of the strains, while the *msrA* gene was observed in 11% isolates. Eighty-five percent of the strains were described as biofilm-positive by phenotypic methods and carried the *icaADB* gene cluster. Multidrug resistance and the biofilm-forming ability in most of the strains tested may contribute to antimicrobial therapy failure (*p* < 0.05).

## 1. Introduction

Hospital-acquired infections are among the major challenges in current epidemiology and public health [1]. Gram-positive cocci, especially *Staphylococcus aureus* and *Staphylococcus epidermidis* resistant to methicillin (methicillin-resistant *S. aureus* (MRSA); methicillin-resistant *S. epidermidis* (MRSE)), have been increasingly prevalent etiological factors of nosocomial infections worldwide [2].

Since *S. epidermidis* colonizes human skin and mucosa during the first few hours of life, after 24 h, skin of most healthy neonates (84%) is colonized by this bacterial species [3]. Furthermore, *S. epidermidis* can cause opportunistic infections in mature and premature neonates. Infants with low birth weight, immature immune system, and/or damaged skin and mucous membranes are particularly vulnerable. Serious clinical conditions requiring catheterization (venous, urinary, or umbilical), mechanical ventilation, tracheal intubation, parenteral nutrition, or other medical procedures may enhance the risk of infection and sepsis [4,5]. A variety of equipment used in the modern therapy of newborns such as respirators, infusion pumps, monitoring devices, thermometers, stethoscopes, or suction devices may contribute to the spread of the bacteria [6,7].

Premature or sick newborns are mainly treated at neonatal intensive care units (NICU). Hospitalization for more than twenty days is a risk factor for neonatal sepsis, osteomyelitis, dermatitis, necrotizing enterocolitis, and surgical wound infections [8]. The main etiological agents of these infections are coagulase-negative staphylococci, which are frequently multidrug-resistant. Virulence factors of these bacteria enable them to escape the host’s immune response and facilitate adhesion to host tissues and foreign materials [9]. One of the most important *S. epidermidis* virulence factors is extracellular secretion of mucus which facilitates bacterial adhesion to biomaterials, i.e., to the plastic materials used to manufacture medical devices, such as prostheses and catheters [10]. Microorganisms attached to solid surfaces and surrounded by self-produced extracellular polymeric substances (EPS) constitute structurally and functionally complex communities known as the biofilm. As the biofilm develops, bacterial cells aggregate to form microcolonies immersed in the extracellular matrix (ECM) incised with a network of canals containing exopolysaccharides and other organic compounds, e.g., proteins, teichoic acids, nucleic acids, and phospholipids [11]. The resistance of biofilms to toxic substances is likely to be caused by their complex structure, especially by the exopolysaccharide content of the extracellular matrix. EPS form a gel-like substance that acts as a physical barrier against the penetration of antimicrobial agents into bacterial cells [12]. Biofilms prevent penetration of phagocytes, antibodies, and antibiotics into bacterial cells thus increasing their resistance to drugs and disinfectants. Furthermore, metabolism of biofilm-forming bacteria is slowed down and they undergo phenotypic changes determining their resistance and virulence. Moreover, mechanical disruption of the biofilm structure can lead to the dispersal of aggregated bacteria into the bloodstream thus causing dissemination of infection [13].

Methicillin-resistant *S. epidermidis* (MRSE) strains pose an essential threat to hospitalized patients. This mechanism of resistance is associated with the acquisition of the *mecA* gene encoding a specific penicillin-binding protein (PBP2a) and characterized by substantially lower affinity to β-lactam antibiotics [14]. Besides β-lactam resistance, clinical MRSE strains may also acquire resistance to other groups of antimicrobial agents (including aminoglycosides, macrolides, fluoroquinolones, or tetracyclines) used in the therapy of staphylococcal infections. Another clinically important mechanism present among *S. epidermidis* strains includes resistance to macrolides, lincosamides, and streptogramins B (MLS_B_) [15]. This mechanism is associated with target site modification, efflux pumps, and enzymatic modification of antibiotics. The most prevalent mechanism is related to target site modifications of ribosomes by methylases encoded by *erm* genes and results in the resistance to all MLS_B_ antibiotics. Furthermore, the resistance only to 14 and 15-membered macrolides and streptogramins B is associated with *msr*-encoded efflux pumps [16]. The third mechanism is related to enzymatic inactivation of lincosamides [17].

While *Staphylococcus epidermidis* may be the causative agent of severe hospital-acquired infections in some clinical cases, it is difficult to distinguish between the infection and contamination of the clinical specimen. It has been noted that the strains causing infections are more likely to carry genetic determinants of resistance, pathogenicity, and biofilm, but the results obtained so far suggest that there is no single distinction marker which can be used in routine diagnostics. Therefore, the studies concerning the analysis of *Staphylococcus epidermidis* virulence, resistance, and biofilm formation contribute to enhancing the knowledge concerning this important bacterium [18,19].

The aims of the study included the analysis of (1) antimicrobial susceptibility, (2) selected resistance phenotypes and genotypes, (3) biofilm formation (among the group of clinical *S. epidermidis* strains isolated at the University Children’s Hospital in Krakow), and (4) the evaluation of the biofilm detection method. Therefore, the study of the phenotype and the genotypic characterization of virulence of clinical *S. epidermidis* isolates is of great value in understanding their roles in the pathogenesis.

## 2. Results

### 2.1. Species Identification of the Tested Strains

**Figure 1:**
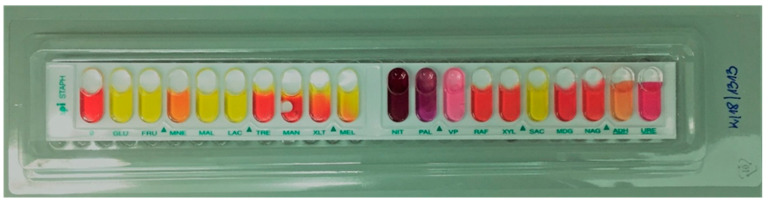
Identification strip of the API Staph system (bioMérieux, Poland). The metabolic processes taking place during incubation change the color of the substrates. The photo shows the identification strip for the clinical strain *S. epidermidis* K/18/1313.

**Figure 2:**
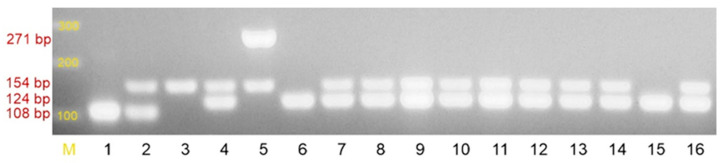
Agarose gel electrophoresis of multiplex PCR performed in order to identify bacterial species and detect the presence of the *mecA* gene: M—DNA molecular weight marker 100 bp (GeneRuler 100 bp DNA Ladder, ThermoFisher Scientific), 1-*. aureus* ATCC 25923 *mecA*(–) strain, 2-*S. aureus* ATCC 43300 *mecA*(+) strain, 3-clinical *mecA*(+) strain, 4- clinical *Staphylococcus epidermidis* strain, 5-*Staphylococcus haemolyticus* ATCC 29970 strain, 6–*Staphylococcus epidermidis* ATCC 12228 strain, 7-16-clinical *Staphylococcus epidermidis* strains. List of expected amplicons for (1) species identification: *S. aureus*-108 bp; *S. epidermidis*-124 bp; S. *haemolyticus*-271 bp; (2) *mecA* gene-154 bp.

The species *S. epidermidis* was confirmed in phenotypic (classical microbiology identification methods and the biochemical API Staph test (bioMérieux, Poland, Figure 1)) and genetic studies (the presence of a 124-bp amplicon-specific fragment for *S. epidermidis* species, Figure 2) of all the tested strains [20]. The identification of *S. epidermidis* was confirmed, while 89/100 samples were *mecA*(+) (Supplementary Material Table S1).

### 2.2. Resistance to Methicillin

The resistance to methicillin was tested in parallel using the disk diffusion (30 µg cefoxitin) and multiplex PCR techniques (the presence of a *mecA* amplicon of 154 bp) [20]. Of the 100 tested strains, 89% confirmed both methicillin resistance (MRSE) by the disk diffusion method and presence of the *mecA* gene (Figure 2, Supplementary Material Table S1).

### 2.3. Macrolide, Lincosamide, and Streptogramin (MLS) Resistance Mechanisms

The phenotypic method showed that 53 (53%) isolates had the constitutive resistance phenotype (cMLS_B_), and three (3%) had the inducible resistance phenotype (iMLS_B_). The strains demonstrating either constitutive or inducible mechanism of resistance to macrolides, lincosamides, and streptogramins B should be reported as resistant to clindamycin. The MS_B_ phenotype was identified in 11 (11%) strains so neither macrolides (comprising a 14- or 15-membered ring) nor streptogramins B should be used as therapeutic agents.

**Figure 3:**
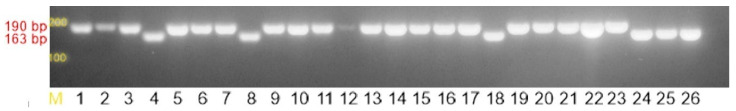
Agarose gel electrophoresis of multiplex PCR products determining the resistance to macrolides, lincosamides, and streptogramins B: *ermC* (190 bp), *msrA* (163 bp). M-DNA molecular weight marker 100 bp (GeneRuler 100 bp DNA Ladder, ThermoFisher Scientific), 1–25-linical strains of *Staphylococcus epidermidis*, 26-*Staphylococcus aureus* ATCC BAA-976 *msrA*(+) strain.

Two separate PCR assays were used to detect erythromycin resistance genes: the first one detected the *ermA, ermC*, and *msrA* genes while the second assay identified the *ermB* and *mef* genes [21]. The *ermC* gene (amplicon size of 190 bp) was detected in 56 (56%) strains in which the presence of the MLS_B_ (either constitutive and inducible) resistance mechanism was detected by phenotypic testing, whereas the 11 (11%) strains previously identified as MS_B_ carried the *msrA* gene (product size of 163 bp) encoding an efflux pump, a member of the ATP-dependent membrane superfamily of transporters (ABC transporters) (Figure 3, Supplementary Materials).

**Figure 4:**
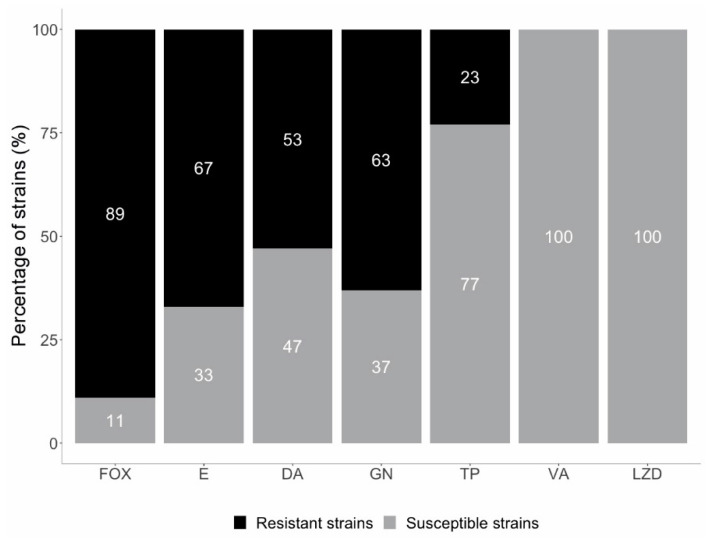
Susceptibility of the tested *S. epidermidis* strains (FOX—cefoxitin, E—erythromycin, DA—clindamycin, GN—gentamicin, TP—teicoplanin, VA—vancomycin, LZD—linezolid).

According to the disk diffusion test, 63 (63%) strains were resistant to gentamicin. All isolates were susceptible to linezolid (*n* = 100). Furthermore, it was confirmed that all the tested *S. epidermidis* strains were susceptible to vancomycin with the MIC values ranging from 0.25 to 3 μg/mL. Resistance to teicoplanin was found in 23 (23%) of the *S. epidermidis* isolates with the MIC values ranging from 6 to 16 μg/mL (Figure 4, Supplementary Material Table S1). No intermediate susceptible strains were found.

### 2.4. Biofilm-Forming Capacity

**Figure 5:**
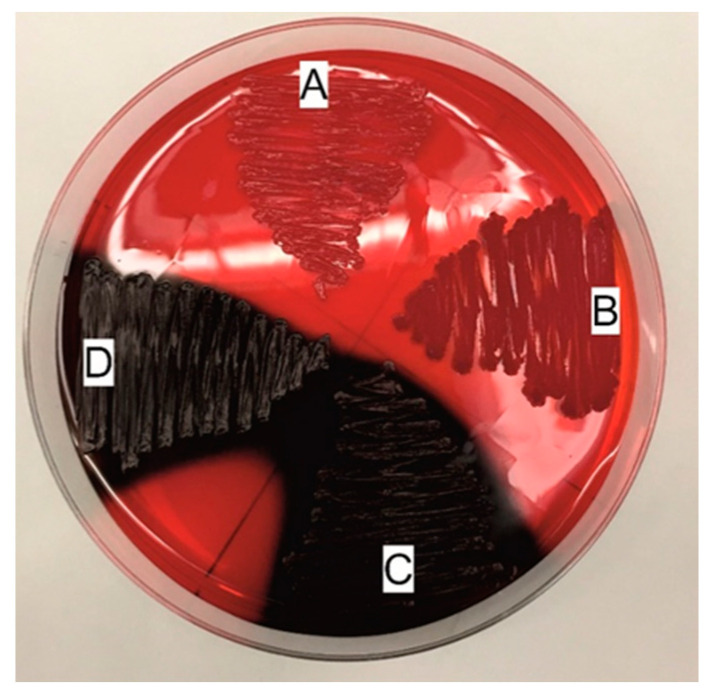
Freeman method—qualitative evaluation of the biofilm-forming capacity of the bacteria cultured on a Congo Red-containing medium. A—non-biofilm producer *Staphylococcus epidermidis* ATCC 12228 (the negative control), B—non-biofilm producer K/18/313 (clinical strain), C—biofilm producer *S. epidermidis* ATCC 35984 (the positive control), D—biofilm producer K/12/8915 (clinical strain).

According to the results obtained using the method described by Freeman [22], 86% (*n* = 86) of the *S. epidermidis* strains formed a biofilm. The biofilm-positive strains produced black, usually matte colonies when grown on the Congo Red supplemented medium. The remaining 14% (*n* = 14) of the strains were unable to form a biofilm; they formed intense red colonies (Figure 5, Supplementary Material Table S1).

The strains which produced a biofilm outnumbered the strains which were unable to form a biofilm (χ^2^ = 49.00, *p* < 0.001).

Furthermore, when Christensen (quantitative) method [23] was applied, the results showed that 86% (*n* = 86) of the tested strains were strong biofilm producers. Moderate and weak biofilm producers comprised 3% (*n* = 3) and 11% (*n* = 11), respectively. The strains were classified (Table 1, Supplementary Material Table S1). It is worth noting that in three (3%) of the tested strains, Christensen method confirmed the production of a biofilm with medium intensity, while when Freeman method was used, the strains grew red, and the presence of the *ica*ADB gene cluster was not confirmed. Staining with crystal violet showed that the studied strains rapidly formed biofilms in vitro (Table 1).

The strains characterized by resistance mechanisms such as MLS_B_, MS_B_, as well as resistance to methicillin, gentamicin, and teicoplanin had a strong ability to produce a biofilm (as shown in Table 2 and Table 3).

The group of strains with methicillin resistance mechanisms outnumbered the group without such a mechanism, χ^2^ = 60.84, *p* < 0.001. The strains with another type of mechanism (MLS_B_, MS_B_) outnumbered the strains without such a mechanism, χ^2^ = 26.48, *p* < 0.001.

Additionally, when we compared the numbers of strains with both types of the resistance mechanism, we statistically proved that groups with MRSE + MLS_B_ or MS_B_ were the largest, χ^2^ = 122.6, *p* < 0.001.

We obtained more strains resistant to gentamicin (χ^2^ = 6.76, *p* < 0.01) and teicoplanin (χ^2^ = 29.16, *p* < 0.001) than without the ability to resist to both antibiotics.

### 2.5. Application of Logistic Regression to Predict A Dichotomous Variable for Three Biofilm Incidence Study Models

We also tested if we could predict biofilm formation (dependent variable) based on a few independent variables (types of resistance mechanisms, resistance to gentamicin and teicoplanin). To check these assumptions, we ran the logistic regression on the presented data (86 observations). We did not have to exclude any outliers, and the variables did not show any collinearity. The statistical analysis concerning the simultaneous impact of the independent variables on biofilm formation demonstrates that none of the above regressors was statistically significant. Consequently, types of resistance mechanisms, resistance to gentamicin and teicoplanin, cannot help in predicting the appearance of a biofilm.

The statistical analysis concerning the simultaneous impact of resistance mechanisms, resistance to gentamycin and teicoplanin, as well as the presence of a biofilm confirmed by three methods showed that none of the regressors above was statistically significant.

For Freeman method of biofilm formation, the chi-squared test indicated that the tested model was statistically insignificant, χ^2^ = 4.61, *p* = 0.466 (Table 4).

For Christensen method of biofilm formation, the chi-squared test indicated that the tested model was not statistically significant, *χ*^2^ = 2.81, *p* = 0.73. For the results, see Table 5.

For the genetic method of biofilm formation, the chi-squared test indicated that the tested model was statistically insignificant, χ^2^ = 4.61, *p* = 0.466. For the results, see Table 6.

Please note that the results for logistic regressions for Freeman method and the genetic method were the same. Both methods gave exactly the same results for biofilm marking. Freeman method, although easier and faster to perform than the *ica*ADB gene cluster detection, can be recommended as a screening test for identifying biofilm production by *S. epidermidis* strains.

**Figure 6:**
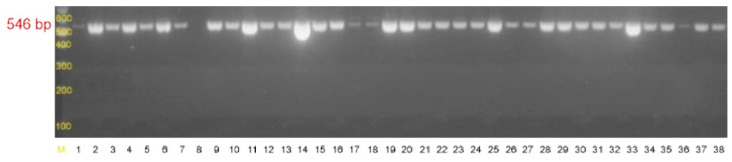
Agarose gel electrophoresis of PCR products of the *ica*ADB gene with the size of 546 bp. M-DNA molecular weight marker 1000 bp (DNA Marker 1, A&A Biotechnology Poland), 1-*S. epidermidis* ATCC 35984 *ica*ADB(+) strain, 2–28-clinical strains of *Staphylococcus epidermidis*.

PCR analyses showed that 86 out of the 100 strains carried the *ica*ADB gene cluster [24] which gave a product of 546 bp (Figure 6, Supplementary Material Table S1).

## 3. Discussion

*Staphylococcus epidermidis* is the third most significant pathogen associated with hospital-acquired infections. In Poland, this bacterium is the main pathogen responsible for bloodstream infections in neonatal units [25,26]. Resistance of *S. epidermidis* strains to methicillin is a significant clinical problem associated with the expression of the *mecA* gene encoding altered penicillin-binding protein PBP2a (PBP2′) [14,27]. Brzychczy-Włoch et al. [28], who analyzed 63 *S. epidermidis* strains, obtained similar results: 98% of the strains were methicillin-resistant. The research of Al-Mulla et al. [29] revealed the presence of 26 *S. epidermidis* clinical strains isolated from children staying at hospital departments of hematology and oncology, and they also demonstrated a high percentage of methicillin-resistant isolates (77%). In another study by Najar-Peerayeh et al. [30], 92.2% of the investigated strains isolated from intensive care patients were resistant to methicillin. Chaieb et al. [21] examined 33 clinical *S. epidermidis* strains and revealed that 78.1% of the isolates carried the *mecA* gene. Notably, the rate of MRSE strains (89%) found in our study was within the range reported globally (75–90%) [31].

Resistance to erythromycin was observed in 67% of the tested strains. Similar results were obtained by Szczuka et al. [32] who classified 76% of the studied strains as erythromycin-resistant. On the other hand, Wojtyczka et al. [33] reported a two times smaller number of erythromycin-resistant strains (43.7%), whereas Brzychczy-Włoch et al. [28] found 84% of the tested strains to be erythromycin-resistant. Of the 100 analyzed isolates, 40% had the constitutive resistance phenotype (cMLS_B_), 27% had the inducible resistance phenotype (iMLS_B_), and 11% were identified to have the MS_B_ resistance phenotype. Similar results were reported by Szczuka et al. [32] who observed that more than half of the analyzed isolates expressed the MLS_B_ phenotype of resistance, and most of them—the constitutive phenotype, whereas in the study by Brzychczy-Włoch et al. [28], the constitutive phenotype of resistance (cMLS_B_) was found in 43% of the strains, inducible phenotype (iMLS_B_)—in 16%, and MS_B_—in 40% of the analyzed isolates. Juda et al. [34] reported the constitutive phenotype of resistance (cMLS_B_) in 36%, inducible phenotype (iMLS_B_)—in 18.7%, and MS_B_—in 45.3% of the studied strains.

The occurrence of genes responsible for erythromycin resistance was confirmed by two independent multiplex PCR reactions (respectively, *ermA*, *ermC*, *msrA*, *ermB*, and *mef* genes). The *ermC* gene appeared to be the most prevalent among the analyzed *S. epidermidis* isolates. Similar results were obtained by Chaieb et al. [21], Brzychczy-Włoch et al. [28], and Juda et al. [34] who reported the predominance of the *ermC* and *msrA* genes and did not observe any formation of the *mef* gene product. In the publication by Szemraj et al. [35], the MLS_B_ resistance mechanism was common in *S. hominis, S. haemolyticus*, and *S. epidermidis* isolates—respectively, in 100%, 78%, and 59% of the isolates. In most cases, it was the constitutive type. The type of the *erm* gene also depends on the geographical region of isolation. For example, *ermC* was previously detected in 50% of the strains exhibiting the MLS_B_ resistance in Great Britain, whereas it was detected in 90% of those originating from Denmark. The distribution of *erm* genes depends on the bacterial species [36].

Sixty-three percent (*n* = 63) of the strains proved to be gentamicin-resistant. Al-Mulla et al. [29] found that 23% of the analyzed strains were resistant to gentamicin, whereas Szczuka et al. [32] reported a higher percentage of resistance (47%). Other authors obtained even higher results, such as Brzychczy-Włoch et al. (93%) [28] and Białkowska-Hobrzańska et al. (85%) [37]. New breakpoints for aminoglycosides were published in the 2021 EUCAST clinical breakpoint tables (v 11.0). In systemic infections, aminoglycosides must be used in combination therapy. In such situations, the breakpoints in parentheses can be used to distinguish organisms with acquired resistance mechanisms from wild-type strains without resistance mechanisms [38].

Vancomycin is mainly used to treat severe or complicated infections caused by multiresistant bacteria including *Staphylococcus* spp., *Enterococcus* spp., and *Streptococcus* spp. (*Streptococcus pneumoniae*). In clinical practice, vancomycin is effective in the therapy of skin and soft tissue infection, pneumonia, urinary infection, endocarditis, prosthetic device-associated infection, blood stream infections, and other systemic infections. Resistance to teicoplanin accompanied by susceptibility to vancomycin might be explained by incomplete cross-resistance which is a common feature of the abovementioned group of antibiotics. It should be noted that teicoplanin exhibits a lower bactericidal activity against *S. epidermidis* [39]. All the tested isolates were susceptible to vancomycin (MIC < 4 μg/mL), but 23% of these strains were resistant to teicoplanin (MIC > 4 μg/mL). Tevell et al. [40] and Najar-Peerayeh et al. [30] also reported 100% susceptibility to vancomycin, but only 11.5% strains were teicoplanin-resistant in their studies. The results obtained by Brzychczy-Włoch et al. [28] and Tevell et al. [40] were similar (100% susceptibility to vancomycin and 13% resistance to teicoplanin). The results reported by Al-Mulla et al. [29] were much the same (23% of the strains resistant to teicoplanin in the absence of vancomycin-resistant isolates).

Researchers do not agree about the effect of biofilm-forming capacity on the pathogenicity of staphylococcal strains. Some of them consider biofilm to play only a negligible role in the pathogenesis of infection, while others believe that the biofilm-forming capacity distinguishes between the commensal and pathogenic bacteria [41,42].

According to the results obtained by the method of Freeman, 86% of the analyzed *S. epidermidis* strains produced a biofilm and released it extracellularly in contrast to 14% of the strains not producing a biofilm. There is only scarce data on strains isolated from patients of neonatal departments. According to the studies of Grzebyk et al. [43], as much as 90% of the *S. epidermidis* strains produced a biofilm, which was demonstrated using Freeman method. Other authors reported that, on average, 71–73% of the *S. epidermidis* strains exhibited mucoid colony morphologies when grown on the Congo Red agar [44,45]. A study of biofilm production by Christensen method indicated that 90% of the *S. epidermidis* strains produced a biofilm, of which 87% were strong biofilm producers and 3% were moderate biofilm producers. Similar results were obtained by the teams of Indian [46] and Latvian researchers [47].

Of the 100 analyzed isolates, 86 carried the *ica*ADB genes encoding the polysaccharide intercellular adhesin. Similar results were obtained by Līduma et al. [47], whereas Cafiso et al. [45] and Oliveira et al. [44] reported that only 45% of the analyzed strains carried the *ica*ADB gene cluster. Consistent results were obtained using the abovementioned methods.

In our research, 3% of the strains described as moderate biofilm producers by Christensen (quantitative) method did not carry the *ica* gene and gave a negative result on the Congo Red agar. Biofilm-forming *S. epidermidis* strains in which the genes for the *ica* operon were not found have been described by Fraiha et al. [48,49]. It is considered that the role is taken over by accumulation-associated protein *aap* and autolysin *atl*E [50,51].

Since recently, a new concept of biofilm-related disease has emerged in medical science [52]. Biofilm-related diseases result from infections associated with the use of medical implants, chronic infections in which biomaterials are not involved, and even malfunction of life-supporting equipment [53]. An example of a significant biofilm role in the course of a disease could be pneumonia in patients with cystic fibrosis. While most studies have been focused on *Pseudomonas aeruginosa* infections, relatively fewer articles have attempted to understand the role of a *Staphylococcus aureus* biofilm in cystic fibrosis. Pietruczuk-Padzik et al. [54] demonstrated that *Staphylococcus aureus* strains isolated from the respiratory tract of cystic fibrosis patients are capable of forming a biofilm in vitro and may cause chronic respiratory infections. The aim of the study was to evaluate two screening methods for detecting biofilm formation by eighty clinical *Staphylococcus aureus* isolates from cystic fibrosis patients and to evaluate biofilm production in 96-well polystyrene tissue culture plates depending on the culture medium used (Luria–Bertani broth (LB), tryptic soy broth supplemented with 2% glucose (TSBglu), and brain heart infusion (BHI)).

## 4. Materials and Methods

### 4.1. Phenotypic Methods

#### 4.1.1. Bacterial Strains

One hundred *S. epidermidis* nonduplicate isolates from the blood (*n* = 96), cerebrospinal fluid (*n* = 3), and dialysis fluid (*n* = 1) of children hospitalized at the following departments, Neonatal Pathology (*n* = 89), Infant Department (*n* = 5), and Pediatric Surgery (*n* = 6), were selected for the study. Inflammation markers, i.e., white blood cell count, CRP (C-reactive protein) value, platelet count (PLT), procalcitonin index, as well as leading clinical diagnosis, information about the presence of a catheter, intubation, birth weight, date of sample collection or date of admission to the ward, and the presence of resistance mechanisms were the initial criteria for selecting the research group. The samples were collected between 2015 and 2018. These isolates were identified to the species level using classical microbiology identification methods and the biochemical API Staph test (bioMérieux, Poland). The study was approved by the Bioethics Committee of the Jagiellonian University (No. KBET/263/B/2013).

#### 4.1.2. Antimicrobial Susceptibility Testing

To determine the drug resistance phenotype, the Kirby–Bauer disk diffusion method was used with the Mueller–Hinton agar (bioMérieux, Poland), 0.5 McFarland inoculum, and the following antibiotics (Oxoid-Argenta, Poland): cefoxitin (30 μg), erythromycin (15 μg), clindamycin (2 μg), gentamicin (10 μg), and linezolid (10 μg) were used in the study. Zones of inhibition were measured after the growth of bacteria overnight (18 ± 2 h) at 35 ± 2 °C. *S. aureus* ATCC 29213 was used as the quality control.

In order to identify the methicillin (MRSE) and macrolide, lincosamide, and streptogramin B resistance (MLS_B_) among the tested strains, cefoxitin (30 μg), erythromycin (15 μg), and clindamycin (2 μg) disks were used.

The MLS_B_ phenotype can take two forms: (1) constitutive (cMLS_B_) or (2) inducible (iMLS_B_). Strains with the constitutive MLS_B_ resistance phenotype (cMLS_B_) show resistance to erythromycin and clindamycin while strains with the induced MLS_B_ resistance phenotype (iMLS_B_) show resistance to erythromycin and susceptibility to clindamycin with a characteristic D-zone, and strains with MS_B_ are resistant to erythromycin and sensitive to clindamycin.

In the case of both an inductive and constitutive mechanism, macrolides, lincosamides, and streptogramins B should not be used in therapy. Detection of the MS_B_ mechanism excludes the use of 14- and 15-membered macrolides and streptogramins B.

Susceptibility to vancomycin and teicoplanin was additionally tested in the MRSE strains and the minimal inhibitory concentration (MIC) values were determined by the Etest method (bioMérieux, Poland). A series of twofold dilutions of an antibiotic are incorporated on a plastic carrier strip from which the antibiotic diffuses freely into the agar, creating a diffusion gradient along the length of the strip. After incubation overnight, the MIC is read as the point where the growth inhibition ellipse intersects the MIC scale on the strip.

The results were interpreted according to the European Committee on Antimicrobial Susceptibility Testing (EUCAST) clinical breakpoint tables 2021 v 11.0 and EUCAST Disk Diffusion Test Methodology v 9 [38,55].

#### 4.1.3. Biofilm Detection

Evaluation of the biofilm-forming capacity was carried out on all the *S. epidermidis* isolates selected using two different phenotypic methods and the genetic method.

##### Freeman Method

The studied strains were cultured on the tryptic soy agar (TSA, bioMérieux, Poland), supplemented with 36 g/mL saccharose and 0.8 g/L Congo Red (Sigma Aldrich, Poland). After 24 h of aerobic incubation at 37 °C, the cultures were kept at room temperature for another 24 h, and then the colony morphology was determined. A positive result was indicated by black matte or blackish-brown matte colonies, whereas a negative result was recorded when strains developed red mucoid colonies [22]. Reference strains represented by *S. epidermidis* ATCC 35984 and *S. epidermidis* ATCC 12228 were used as the positive and negative controls, respectively.

##### Christensen Method

One hundred ninety microliters of the brain heart infusion broth (BHI, bioMérieux, Poland) containing 1% saccharose and 10 μL of 18-h bacterial cultures were added to each well of polystyrene 96-well microtiter plates (Nest Scientific Biotechnology, USA). After 48-h incubation, the wells were washed with sterile saline solution and dried at 37 °C for 2 h. Next, the plates were stained with crystal violet and the biofilm-bound dye was extracted with 96% ethanol [23]. The extracts were transferred to another 96-well plate followed by optical density (OD) measurements at 600 nm (Sunrise, Tecan Biotech, Poland). The results were interpreted taking into account the results of optical density (OD) measurements of the biofilm. The experiment was performed in triplicate and repeated three times. The cutoff value (ODc) discriminating biofilm producers from non-biofilm producers was defined as three standard deviations (SD) above the mean OD of the negative control (*Staphylococcus epidermidis* ATCC 12228). The ODc value was calculated for each microtiter plate separately (Table 7). The abovementioned interpretation of biofilm production was based on the criteria presented by Stepanovic et al. [56].

##### Scanning Fluorescent Microscope

**Figure 7:**
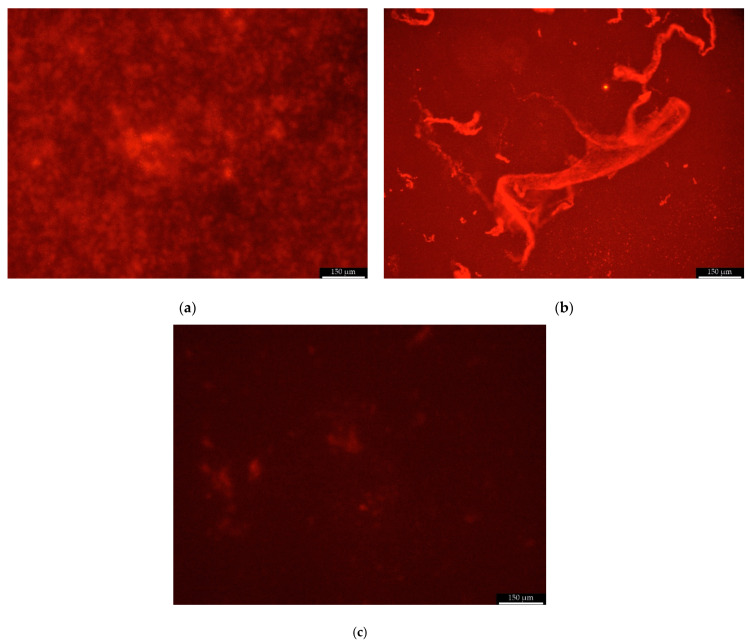
Sample photo of the biofilm formed by clinical strain K/12/8915 stained with FilmTracer ™ SYPRO ™ Ruby Stain and visualized using a scanning fluorescent microscope ((**a**) compact biofilm structure at the bottom of a plate well, (**b**) extracellular mucus on the biofilm surface, and (**c**) *S. epidermidis* ATCC 12228 strain that does not produce a biofilm). Scale bar =150 µm.

The biofilm formed in the wells of the microtiter plate (Nest Scientific Biotechnology, USA) by Christensen method was visualized using a scanning fluorescent microscope Leica DMi8 (Leica Microsystems, Wetzlar, Germany). With the SYPRO Ruby Biofilm Matrix Stain, the excitation/emission wavelengths were 450 nm and 610 nm, respectively. The scanning fluorescent microscope images were analyzed by the image processing software Leica Application Suite (LAS X, Leica Microsystems, Wetzlar, Germany). Biofilms were stained with 200 μL FilmTracer™ SYPRO™ Ruby Stain Biofilm Matrix (ThermoFisher Scientific, Figure 7a,b) per each plate well and incubated in the dark for 30 min at room temperature, then rinsed with distilled water. The preparation of the samples for imaging was performed according to the staining procedure recommended by the manufacturer [57].

### 4.2. Genetic Methods

#### 4.2.1. Identification of the Tested Strains to the Species Level and Detection of Selected Resistance Mechanisms

Genetic analysis included: (1) species identification, (2) detection of the *mecA* gene (associated with methicillin resistance), and (3) analysis of the presence of the *erm*, *msr*, and *mef* gene families (related to the resistance to macrolides, lincosamides, and streptogramins B).

Bacterial genomic DNA was isolated using the Genomic Mini Kit (A&A Biotechnology, Poland) according to the manufacturer’s protocol.

The next step of the study included the multiplex PCR reaction performed in order to identify the bacterial strains to the species level and to detect the presence of the *mecA* gene. The following primers presented in Table 8 were used to amplify the fragments specific for *S. aureus* (SA1, SA2), *S. epidermidis* (SE1, SE2), *S. haemolyticus* (SH1, SH2), and the *mecA* gene (MRS1, MRS2) [20]. DNA amplification was performed using a Biometra T Personal thermocycler (Biometra, Germany) with the following thermal profile: initial denaturation at 94 °C for 2 min, next 30 cycles of the following profile: denaturation at 94 °C for 2 min, primer binding at 56 °C for 1 min, extension at 72 °C for 1.5 min, and final extension at 72 °C for 5 min.

The genes encoding macrolide resistance (*ermA, ermB, ermC, msrA* and *mef*) were detected using multiplex PCR under the following conditions: initial denaturation at 94 °C for 3 min, 30 cycles under the following conditions: 94 °C for 30 s, 55 °C for 30 s, 72 °C for 2 min, and final extension at 72 °C for 2 min [21]. The products of both PCR reactions were detected by electrophoresis on a 2% agarose gel (MAXIMUS, Polskie Agarozy, Poland) containing ethidium bromide (Sigma Aldrich, USA) performed at the constant voltage of 90 V for 90 min and were visualized using a UV transilluminator (ECX 20M, Vilber Lourmat, France). The assays described above were conducted using the following reference strains as controls: *S. aureus* ATCC 25923, *S. aureus* ATCC 43300, *S. epidermidis* ATCC 12228, *S. aureus* ATCC BAA-976, and *S. haemolyticus* ATCC 29970 (Table 9).

#### 4.2.2. Detection of Genes Associated with Biofilm Formation

Detection of the *ica*ADB gene cluster (Table 10) [24] was carried out in a thermocycler (Biometra, Germany) under the following conditions: initial denaturation at 94 °C for 3 min followed by 30 cycles of 1 min denaturation at 94 °C, annealing for 1 min at 58 °C, and extension for 1 min at 72 °C with the final extension step at 72 °C for 5 min. PCR products were detected by electrophoresis on a 2% agarose gel (MAXIMUS, Polskie Agarozy, Poland) containing ethidium bromide (Sigma Aldrich, USA). Five microliters of 1000-bp markers and the PCR products were pipetted to each well (DNA Marker 1, A&A Biotechnology Poland). Electrophoresis was performed at the constant voltage of 90 V for 60 min and the results were visualized using a UV transilluminator (ECX 20M, Vilber Lourmat, France). The gels were documented by a Power-Shot G5 (Canon, Japan) digital camera.

#### 4.2.3. Statistical Analysis

Descriptive statistics were calculated first. Discrete characteristics are expressed as frequency counts and percentages (*n*, %). The data were statistically analyzed using the multivariate logistic regression analysis. Multivariate logistic regression models examined the impact of resistance mechanisms, resistance to gentamycin, and resistance to teicoplanin using three different biofilms methods (separately). The indicators were marked as significant when the *p*-value (two-sided) was lower than 0.05. All statistical analyses were performed using SPSS Statistics version 26 for Macintosh.

For Christensen method, weak and moderate biofilm producers were analyzed as one group and compared to strong producers because of the fact that the sample size was too small for appropriate statistical analysis. Additionally, we combined two types of resistance mechanisms in one variable with six subgroups.

## 5. Conclusions

Due to the clinical and therapeutic problem of multidrug resistance of *Staphylococcus epidermidis* strains isolated from the blood of neonates, it is necessary to conduct clinical analyses to determine their ability to produce a biofilm which may pose an unusual mechanism of resistance. Test extension in order to detect a biofilm should be applied in the case of isolation of multidrug-resistant strains which do not constitute contamination of the collected materials and in the case of therapeutic failures. The results of the research indicate the need in the introduction of biofilm detection methods into the routine diagnostic procedures in clinical microbiology. Our study confirmed that Freeman method is a reliable, less time-consuming, and cost-effective biofilm detection assay.

## Figures and Tables

**Table 1:** Biofilm production by *Staphylococcus epidermidis* isolates according to Christensen method.

Average OD Value	*N* (%)	Biofilm Formation
OD ≤ 0.09	0 (0%)	No
0.09 < OD ≤ 0.18	11 (11%)	Weak
0.18 < OD ≤ 0.36	3 (3%)	Moderate
0.36 < OD	86 (86%)	Strong
	* ODc = 0.09	

* Optical density cutoff value (ODc) = average OD of the negative control + 3×standard deviation (SD) of the negative control (*Staphylococcus epidermidis* ATCC 12228 ODc = 0.06 + 3 × 0.01 = 0.09); N-overall frequency; %-percentage.

**Table 2:** Comparison of resistance to methicillin, macrolides, lincosamides, and streptogramins with biofilm determination using different methods within the analyzed group of *Staphylococcus epidermidis* strains obtained from pediatric patients.

Resistance Mechanisms	Biofilm Formation						
	Christensen Method *N* (%)			Freeman Method *N* (%)		*ica*ADB Gene Cluster *N* (%)	
	Strong	Moderate	Weak	Black	Red	Presence	Absence
MRSE	22 (79%)	1 (3%)	5 (18%)	22 (79%)	6 (21%)	22 (79%)	6 (21%)
MRSE + MLS_B_	48 (91%)	2 (4%)	3 (5%)	48 (91%)	5 (9%)	48 (91%)	5 (9%)
MRSE + MS_B_	7 (88%)	0	1 (12%)	6 (75%)	2 (25%)	6 (75%)	2 (25%)
MLS_B_	1 (100%)	0	0	1 (100%)	0	1 (100%)	0
MS_B_	4 (100%)	0	0	4 (100%)	0	4 (100%)	0
None	4 (67%)	0	2 (33%)	4 (67%)	2 (33%)	4 (67%)	2 (33%)
Total	86 (86%)	3 (3%)	11 (11%)	85 (85%)	15 (15%)	85 (85%)	15 (15%)

MRSE—methicillin-resistant *Staphylococcus epidermidis*; MLS_B_-macrolides, lincosamides, and streptogramins B; MS_B_-macrolides and streptogramins B; N-overall frequency; %-percentage.

**Table 3:** Comparison of resistance to gentamycin and teicoplanin of *Staphylococcus epidermidis* strains obtained from pediatric patients with biofilm determination using different methods.

Biofilm Formation							
Antibiotics	Christensen Method *N* (%)			Freeman Method *N* (%)		*ica*ADB Gene Cluster *N* (%)	
	Strong	Moderate	Weak	Black	Red	Presence	Lack
Gentamicin							
R*	57 (91%)	2 (3%)	4 (6%)	56 (89%)	7 (11%)	56 (89%)	7 (11%)
S**	29 (78%)	1 (3%)	7 (7%)	29 (78%)	8 (22%)	29 (78%)	8 (22%)
Teicoplanin							
R	20 (87%)	1 (9%)	2 (4%)	19 (83%)	4 (17%)	19 (83%)	4 (17%)
S	66 (86%)	2 (3%)	9 (12%)	66 (86%)	11 (14%)	66 (86%)	11 (14%)

R*—resistant; S**—susceptible; N—overall frequency; %—percentage.

**Table 4:** Results of logistic regression for the biofilm formation by *S. epidermidis* (Freeman method).

	*B*	*p*-Value	Exp(B)	95% CI
Resistance mechanism 0		0.369		
Resistance mechanism MLS_B_	−0.50	0.573	0.61	0.11–3.47
Resistance mechanism MS_B_	0.43	0.665	1.53	0.22–10.65
Resistance mechanism MRSE	−0.22	0.811	0.80	0.13–4.90
Resistance to gentamycin	−0.68	0.305	0.51	0.14–1.86
Resistance to teicoplanin	0.49	0.486	1.63	0.41–6.47
Constant	1.87	0.148	6.49	

**Table 5:** Results of logistic regression for the biofilm formation by *S. epidermidis* (Christensen method).

	*B*	*p*-Value	Exp(B)	95% CI
Resistance mechanism 0		0.284		
Resistance mechanism MLS_B_	−1.39	0.224	4.01	0.43–37.52
Resistance mechanism MS_B_	−0.57	0.641	1.78	0.16–19.77
Resistance mechanism MRSE	−0.09	0.921	1.09	0.17–6.97
Resistance to gentamycin	−0.91	0.177	2.48	0.67–9.24
Resistance to teicoplanin	0.16	0.831	0.85	0.19–3.78
Constant	3.05	0.044	0.05	

**Table 6:** Results of logistic regression for the *ica*ADB gene cluster.

	*B*	*p*-Value	Exp(B)	95% CI
Resistance mechanism 0		0.369		
Resistance mechanism MLS_B_	−0.50	0.573	0.61	0.11–3.47
Resistance mechanism MS_B_	0.43	0.665	1.53	0.22–10.65
Resistance mechanism MRSE	−0.22	0.811	0.80	0.13–4.90
Resistance to gentamycin	−0.68	0.305	0.51	0.14–1.86
Resistance to teicoplanin	0.49	0.486	1.63	0.41–6.47
Constant	1.87	0.148	6.49	

**Table 7:** Interpretational criteria for Christensen assay.

Average OD Value	Biofilm Production
OD ≤ ODc *	Non-biofilm producer
ODc < OD ≤ 2×ODc	Weak biofilm producer
2×ODc < OD ≤ 4×ODc	Moderate biofilm producer
4×ODc < OD	Strong biofilm producer

* Optical density cutoff value (ODc) = average OD of the negative control + 3×standard deviation (SD) of the negative control.

**Table 8:** The sequences of primers used in the multiplex PCR method performed in order to identify the bacterial species and to detect the *mecA* gene.

**Primer**	**Sequence (5′-3′)**	**Size of the Product**	[20]
SA1	AATCTTTGTCGGTACACGATATTCTTCACG	108 bp	
SA2	CGTAATGAGATTTCAGTAGATAATACAACA		
SE1	ATCAAAAAGTTGGCGAACCTTTTCA	124 bp	
SE2	CAAAAGAGCGTGGAGAAAAGTATCA		
SH1	GGTCGCTTAGTCGGAACAAT	271 bp	
SH2	CACGAGCAATCTCATCACCT		
MRS1	TAGAAATGACTGAACGTCCG	154 bp	
MRS2	TTGCGATCAATGTTACCGTAG		

**Table 9:** The sequences of primers used to detect the genetic determinants of macrolide resistance with the multiplex PCR.

**Primer**	**Sequence (5′-3′)**	**Size of the Product**	[21]
*ermA1*	TATCTTATCGTTGAGAAGGGATT	139 bp	
*ermA2*	CTACACTTGGCTTAGGATGAAA		
*ermB1*	CTATCTGATTGTTGAAGAAGGATT	142 bp	
*ermB2*	GTTTACTCTTGGTTTAGGATGAAA		
*ermC1*	CTTGTTGATCACGATAATTTCC	190 bp	
*ermC2*	ATCTTTTAGCAAACCCGTATTC		
*msrA1*	TCCAATCATAGCACAAAATC	163 bp	
*msrA2*	AATTCCCTCTATTTGGTGGT		
*mef1*	AGTATCTTAATCACTAGTGC	348 bp	
*mef2*	TTCTTCTGGTACAAAAGTGG		

**Table 10:** The sequences of primers used for PCR detection of the genes involved in biofilm formation.

Primer	Sequence (5′-3′)	Size of the Product
*icaADB-F*	TTATCAATGCCGCAGTTGTC	546 bp
*icaADB-R*	GTTTAACGCGAGTGCGCTAT	

## Data Availability

Not applicable.

## Supplementary Materials

The data that supports the findings of this study are available online at https://www.mdpi.com/article/10.3390/pathogens10070877/s1; Table S1: The results of phenotypic and genetic identification of strains, resistance mechanisms and antibiotics/chemotherapeutic agents with zones of inhibition/MIC values and three methods of biofilm detection.
